# Molecular characterization of clinically isolated *Pseudomonas aeruginosa* with varying resistance to ceftazidime-avibactam and ceftolozane-tazobactam collected as a part of the ATLAS global surveillance program from 2020 to 2021

**DOI:** 10.1128/aac.00670-24

**Published:** 2024-09-10

**Authors:** H. Li, A. Oliver, R. K. Shields, S. Kamat, G. Stone, M. Estabrook

**Affiliations:** 1 IHMA, Schaumburg, Illinois, USA; 2 Microbiology Service, Son Espases University Hospital, IdISBa, CIBERINFEC, Palma, Illes Balears, Spain; 3 Department of Medicine, Division of Infectious Diseases, University of Pittsburgh, Pittsburgh, Pennsylvania, USA; 4 Pfizer, Mumbai, India; 5 Pfizer, Groton, Connecticut, USA; Shionogi Inc., Florham Park, New Jersey, USA

**Keywords:** *Pseudomonas aeruginosa*, ATLAS global surveillance, ceftazidime-avibactam, ceftolozane-tazobactam, WGS

## Abstract

Ceftazidime-avibactam (CZA) and ceftolozane-tazobactam (C/T) are important agents for treating multidrug-resistant *P. aeruginosa* infections. In this study, we evaluated the molecular characteristics of 300 globally collected clinical *P. aeruginosa* isolates non-susceptible (NS) to CZA, C/T, or both agents. Isolates were CZA-NS and C/T-NS (*n* = 57), CZA-susceptible (S) and C/T-NS (*n* = 145), or CZA-NS and C/T-S (*n* = 98) selected from the Antimicrobial Testing Leadership and Surveillance (ATLAS) surveillance program from 2020 to 2021. Characterization was by whole-genome sequencing. Analysis was performed to identify β-lactamase genes and mutations that impact efflux regulation, AmpC regulation, and target binding (PBP3). Of the 57 CZA-NS+C/T-NS isolates, 64.9% carried a metallo-β-lactamase (MBL), and a cumulative 84.2% carried any non-intrinsic β-lactamase [i.e., not *Pseudomonas-*derived cephalosporinase (PDC) or OXA-50-like]. Of the 145 CZA-S+C/T-NS isolates, 26.2% carried an extended-spectrum β-lactamase (ESBL) and no carbapenemase, 17.9% carried a serine-carbapenemase, and 42.1% were negative for non-intrinsic β-lactamases. Of 98 CZA-NS+C/T-S isolates, 34.7% carried mutations previously described as causing an upregulation of the MexAB-OprM efflux pump, while only 9.2% carried a non-intrinsic β-lactamase, and no resistance mechanism was identified in 29.6% of these isolates. MBLs were present in most isolates NS to both agents. More than half of the CZA-S+C/T-NS isolates carried serine β-lactamases. The most frequently identified resistance mechanism identified in CZA-NS+C/T-S isolates was a marker indicating the upregulation of MexAB-OprM. No mechanism was identified that is thought to support resistance to these agents in numerous isolates. This may be due in part to the fact that whole genome sequencing (WGS) cannot directly measure gene expression of chromosomal resistance mechanisms.

## INTRODUCTION

*Pseudomonas aeruginosa* is an opportunistic gram-negative pathogen and a common cause of nosocomial infections, primarily respiratory tract infections, including pneumonia (1). This pathogen is difficult to treat because it can present a wide array of antimicrobial resistance mechanisms, both intrinsic and horizontally acquired. Ceftazidime-avibactam (CZA) and ceftolozane/tazobactam (C/T) are a combination of cephalosporin and β-lactamase inhibitor antimicrobial agents that have broad *in vitro* activity against *P. aeruginosa* (2, 3).

Ceftazidime is a third-generation cephalosporin that has activity against gram-negative bacteria, but the activity of this agent is compromised by the rising prevalence of multidrug resistance (MDR) bacteria. Avibactam is a diazabicyclooctane (DBO) β-lactamase inhibitor that restores the activity of ceftazidime against organisms carrying class A, class C, and some class D β-lactamases, but not class B metallo-β-lactamases (MBLs (4). Mutations that expand the substrate profile of the *Pseudomonas-*derived cephalosporinase (PDC, also called AmpC), overexpression of PDC (5, 6), upregulation of efflux pumps (7–11), and MBLs are the avenues of CZA resistance in *P. aeruginosa* (12).

C/T is an antipseudomonal cephalosporin/β-lactamase inhibitor combination. This agent can overcome some common resistance mechanisms in *P. aeruginosa*. Ceftolozane is stable to hydrolysis by PDC that lacks substrate-expanding mutations, and it is not a substrate of resistance-nodulation cell division (RND) efflux pumps carried by *P. aeruginosa* (13). Resistance to C/T can arise from the upregulation of PDC expression (through mutations in the genes *ampD, ampDh2, ampDh3, ampR, dacB,* and *mpl*) (5, 6, 14–16) or mutations in PDC that expand the substrate profile to include ceftolozane (17–20), MBLs (e.g., NDM, VIM, and IMP), serine carbapenemases (e.g., KPC, some variants of GES), and extended-spectrum β-lactamases (ESBLs) (e.g., VEB, PER, and GES) (13).

In this study, 300 *P*. *aeruginosa* clinical isolates from the Antimicrobial Testing Leadership and Surveillance (ATLAS) global surveillance program were characterized to determine what resistance mechanisms were present in isolates presenting different susceptibility profiles for these agents. This set was divided into three phenotypic populations: isolates that were non-susceptible (NS) to C/T (MIC > 4 µg/mL), isolates that were NS to CZA (MIC > 8 µg/mL), and isolates that were NS to both agents.

## MATERIALS AND METHODS

### Bacterial isolates

A random subsample of 300 isolates of *P. aeruginosa* that were CZA- and/or C/T-NS were selected from the ATLAS global surveillance program from 2020 to 2021 (https://atlas-surveillance.com) divided by region. A total of 16,104 *P. aeruginosa* isolates were collected in 2020 and 2021 for ATLAS from the regions of Africa, Asia, Europe, Latin America, Middle East, and South Pacific. A distribution of approximately 25% of the isolates were from each of the following regions: Africa/Middle East, Asia Pacific, Europe, and Latin America. Of those selected, 57 were CZA-NS and C/T-NS, 145 were CZA-susceptible (S) and C/T-NS, and 98 were CZA-NS and C/T-S.

### Antimicrobial susceptibility testing

Antimicrobial susceptibility testing was performed as a part of the ATLAS Global Surveillance Program using the broth microdilution method according to CLSI guidelines (21). All susceptibility interpretations use (22) breakpoints (22). Note that isolates testing with C/T MIC values of 8 µg/mL are intermediate per CLSI 2023 (22) breakpoints and resistant per EUCAST 2023 breakpoints, while isolates testing with CZA MICs of 16 µg/mL are resistant according to both CLSI and EUCAST breakpoints.

### Whole-genome sequencing

Cultured bacterial isolates were pelleted, and DNA isolation was performed using the Qiagen Qiamp DNA Mini Kit. Library preparation was performed using the Illumina DNA Prep kit, and short-read whole-genome sequencing (2 × 150 bp paired-end configuration) was performed on an Illumina Hiseq platform to a calculated coverage depth of 100×.

Genomic assemblies were created using the CLC genomics workbench version 21.0.5 (Qiagen). To identify genes encoding β-lactamases, assemblies were queried using the Resfinder database (updated 2 September 2021) with coverage and identity thresholds set to ≥35% and ≥72%, respectively (23). Genes identified with <100% identity or coverage were evaluated for a variant by pairwise alignment to a reference sequence from the Bacterial Antimicrobial Resistance Reference Gene Database from the National Center for Biotechnology Information (Bioproject 313047). Multi-locus sequence typing (Seemann T, https://github.com/tseemann/mlst, 24) was used to determine relatedness of isolates.

Genes of interest were analyzed by pairwise alignment to the corresponding gene in reference genome PAO1 (accession NC_002516). These genes were screened for the mutations of known impact and gross disruptions (nonsense mutations, frameshifts, insertions or deletions of greater than 20 codons, and ablation of start or stop codons) as described previously (25). Analysis included previously reported mutations and gross disruptions in genes involved in permeability (26), efflux regulation (*nalC*, *nalD*, *mexR*, *mexZ*, *mexS*, *mexT*, and *nfxB*) (7–11), *bla_PDC_* mutation (17–20), *bla_PDC_* regulation (*ampD*, *ampDh2*, *ampDh3*, *ampR*, *dacB*, and *mpl*) (5, 6, 12, 14–16), and target mutation (*ftsI*) (27, 28) (Table S3). Other genes were analyzed that are not necessarily related to resistance to either agent but are involved in metabolism (*galU*) (29–31), virulence (*exoS/U/T/Y*) (32, 33), or hypermutator phenotypes (*mutL/S*) (34–36).

To create phylogenetic trees, BactSNP v1.1.0 (https://github.com/IEkAdN/BactSNP) was used to create pseudogenomes of each of the phenotypic groups of isolates aligned to PAO1. RAxML-NG (37) was used to create the trees from these alignments, and these were visualized using iTOL (38).

## RESULTS

**Fig 1:**
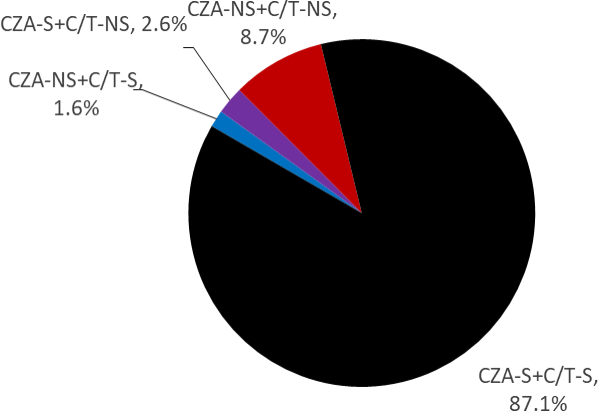
Proportion of collected *P. aeruginosa* isolates, by resistance phenotype (*n* = 16,104).

A total of 16,104 *P. aeruginosa* isolates were collected in 2020 and 2021 for ATLAS. These isolates underwent antimicrobial susceptibility testing by broth microdilution against anti-pseudomonal β-lactam agents (Table 1). By the percentage of susceptible isolates, CZA was the most active agent tested (89.7%) followed closely by C/T (88.7%). Of all isolates, 87.1% were susceptible to both CZA and C/T, 8.7% were NS to both CZA and to C/T, 2.6% were CZA-S and C/T-NS, and 1.6% were CZA-NS and C/T-S (Fig. 1).

**TABLE 1:** *In vitro* activity of anti-pseudomonal β-lactams against all *P. aeruginosa* isolates collected in 2020–2021 (*n* = 16,104)^*a*^

Drug	MIC_50_	MIC_90_	% S	% I/SDD	% R
Cefepime	4	32	78.1	8.4	13.4
CZA	2	16	89.7	0.0	10.3
C/T	1	8	88.7	2.2	9.1
Imipenem	2	>8	62.7	9.9	27.4
Meropenem	0.5	16	74.2	6.0	19.8
Piperacillin-tazobactam	8	>64	72.8	6.9	20.2

^*a*^ I, intermediate; R, resistant; SDD, susceptible, dose dependent (for piperacillin-tazobactam only).

Resistance phenotypes and genotypes categorized by non-intrinsic and intrinsic β-lactamases, indicators for AmpC upregulation, and indicators for efflux upregulation are summarized in Table 2. Of the 57 isolates that are CZA-NS and C/T-NS, 64.9% carried an MBL, and a cumulative 84.2% carried any non-intrinsic β-lactamase. Of 145 CZA-S and C/T-NS isolates, 26.2% carried an ESBL and no carbapenemase, 17.9% carried a serine carbapenemase, and 42.1% did not carry a non-intrinsic β-lactamase. Of 98 CZA-NS and C/T-S isolates, 34.7% carried a mutation previously described as leading to upregulation of the MexAB-OprM efflux pump, while only 9.2% carried a non-intrinsic β-lactamase, and no resistance mechanism was identified in 29.6% of these isolates.

**TABLE 2:** Hierarchical presentation of molecular characteristics of each phenotypic population^*a*^^,^^*b*^

Resistance phenotype		*N*	Resistance genotype (percentage of resistance phenotype)											
			Non-intrinsic β-lactamases				Only intrinsic β-lactamases							
			MBL ± ESBL ± other βla	Serine cpase ± ESBL ± other βla	ESBL ± other βla	Other βla	MexAB-OprM↑ ± other mechanisms	AmpC↑	AmpC↑; MexXY↑	MexCD-OprJ↑	MexEF-OprN↑	MexEF-OprN↑; MexXY↑	MexXY↑	None identified
CZA-**NS**	C/T-**NS**	57	**64.9**	1.8	15.8	1.8	3.5	3.5	1.8	1.8	0.0	0.0	1.8	3.5
CZA-**S**	C/T-**NS**	145	5.5	17.9	**26.2**	8.3	2.8	13.8	2.8	0.0	2.8	0.7	4.1	15.2
CZA-**NS**	C/T-**S**	98	0.0	0.0	1.0	8.2	**32.7**	18.4	3.1	0.0	0.0	0.0	7.1	29.6

^*a*^ [protein name]↑, a genetic marker indicating the increased production of this protein was identified, no direct gene expression assay was performed. Indicators used were: MexAB-OprM↑: *nalC, nalD, mexR*; MexXY↑: *mexZ*; MexCD-OprJ↑: *nfxB*; MexEF-OprN↑: *mexS, mexT;* AmpC↑: *ampD, ampDh2, ampDh3, ampR, dacB, mpl*. Predicted loss of function mutations (except within *ampR* or *mexT*) as well as previously characterized alleles are reported.

^*b*^ Cpase, carbapenemase; S, susceptible; βla, β-lactamase.

**FIG 2:**
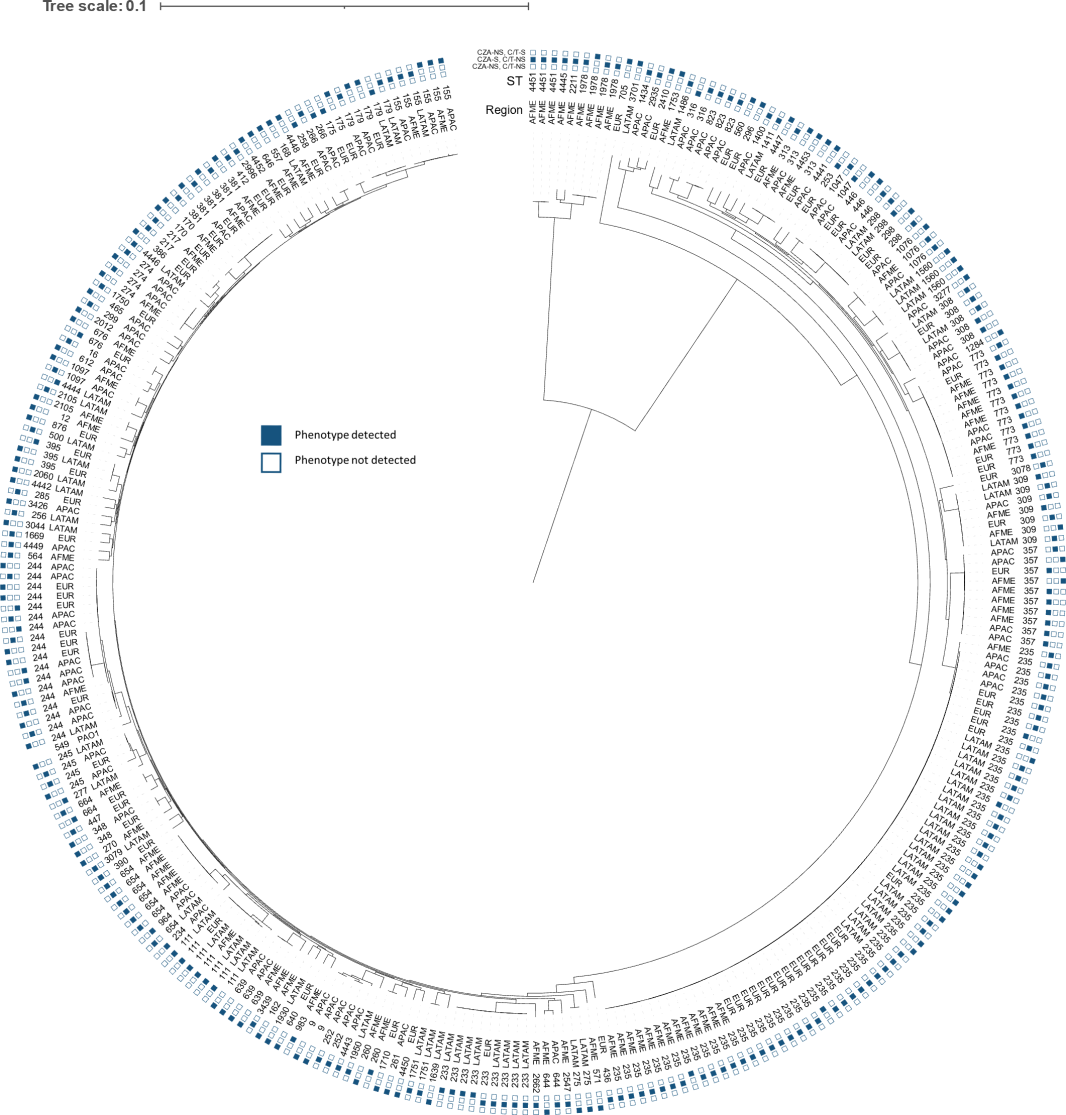
Phylogenetic analysis of all characterized isolates, by region and antimicrobial susceptibility. AFME, Africa/Middle East; APAC, Asia/Pacific; EUR, Europe; LATAM, Latin America, ST, sequence type; CZA, ceftazidime-avibactam; C/T, ceftolozane-tazobactam.

A phylogenetic tree of all characterized isolates and resistance phenotypes shows the distribution of sequence types and region (Fig. 2). The globally disseminated high-risk clone ST235 (*n* = 63) was identified across all regions. In this study, ST235 clones were infrequently CZA-NS, except those collected in Latin America (10/24, 42% NS versus 4/39, 10% NS rest of world). Another high-risk clone, ST244 (*n* = 18), was identified in all regions with variable resistance phenotypes. Two clones, ST773 (*n* = 12) and ST357 (*n* = 10), were most commonly identified among isolates collected in Africa/Middle East and were predominantly NS to both agents (11/12 and 7/10 isolates NS to both agents, respectively). Two clones, ST233 (*n* = 9) and ST111 (*n* = 7), were most frequently identified in Latin America, with all isolates of ST233 testing C/T-NS and ST111 predominantly NS to both agents (6/7 isolates). Finally, ST309 (*n* = 7), ST381 (*n* = 6), and ST155 (*n* = 6) were also identified from various regions and with various resistance phenotypes.

**FIG 3:**
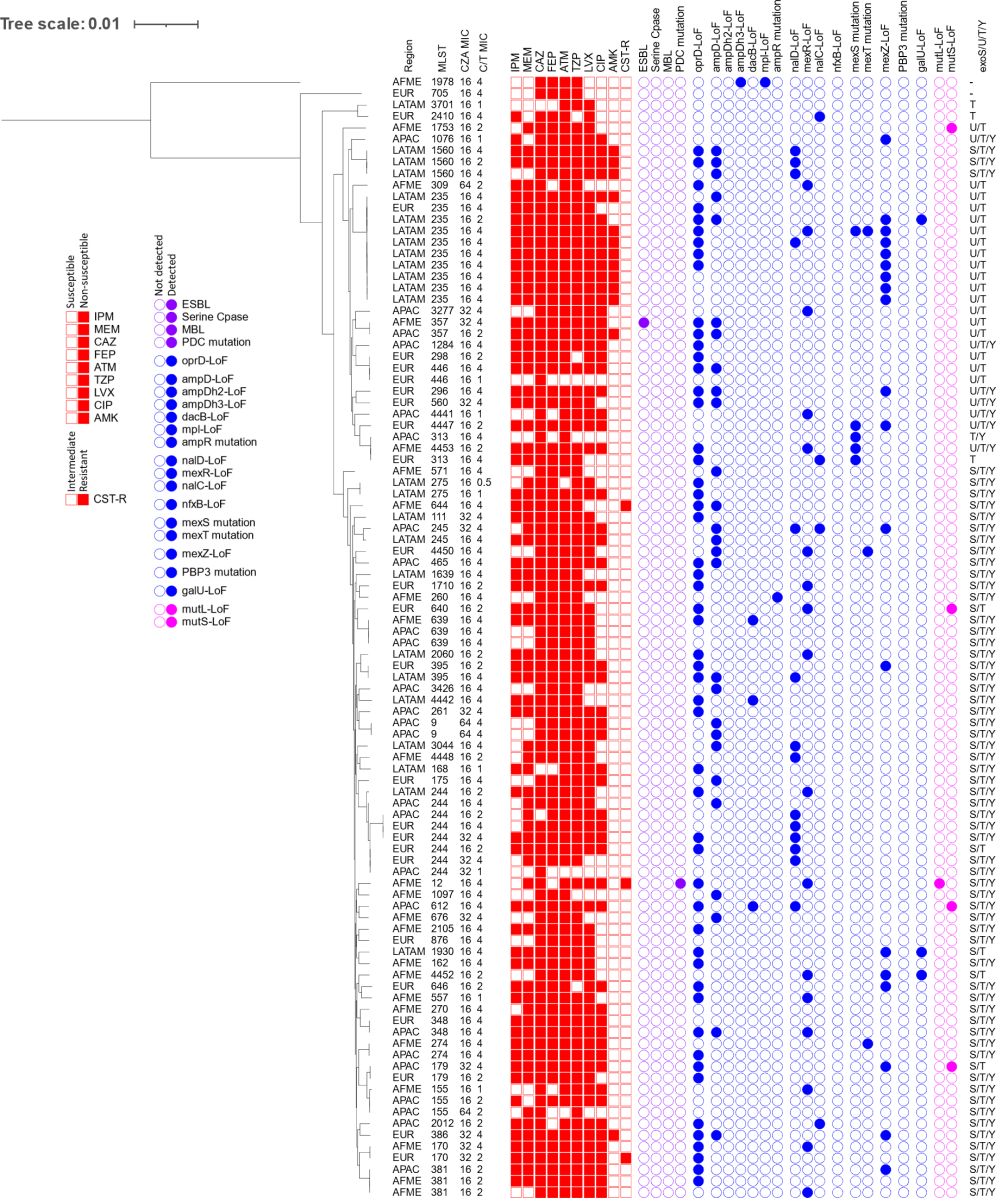
Molecular characteristics and antibiogram of CZA-NS and C/T-S isolates. CZA, ceftazidime-avibactam; C/T, ceftolozane-tazobactam; IPM, imipenem; MEM, meropenem; CAZ, ceftazidime; FEP, cefepime; ATM, aztreonam; TZP, piperacillin-tazobactam; LVX, levofloxacin; CIP, ciprofloxacin; AMK, amikacin; CST-R, colistin-resistant; AFME, Africa/Middle East; APAC, Asia/Pacific; EUR, Europe; LATAM, Latin America; LoF, loss of function. Filled boxes indicate that the isolate tested was NS or susceptible, dose dependent (piperacillin-tazobactam only) to the named agent. Filled circles indicate that the named genotype was identified in that isolate. Colors are used to separate: antimicrobial susceptibility data (red), enzymatic resistance mechanisms (purple), non-enzymatic resistance mechanisms [blue, (left to right: permeability, ampC upregulation, mexAB-oprM, mexCD-oprJ, mexEF-oprN, mexXY, target mutation, and metabolism)], and genes associated with hypermutation (pink).

A phylogenetic tree of CZA-NS and C/T-S isolates with molecular characteristics and antibiogram is presented in Fig. 3. In this population (*n* = 98), the most frequently identified resistance genotypes were regulatory mutations for MexAB-OprM (35%) or PDC (33%). Most of these isolates did not carry an acquired β-lactamase, with one notable exception carrying VEB-1 (1%). Most of these isolates carried the type III secretion effector *exoS*, and a notable number of isolates have a loss-of-function mutation in *exoY*. Two isolates, collected in Africa/Middle East and Europe with sequence types ST1978 and ST705, respectively, have complete absence of *exoS*, *exoU*, *exoT*, and *exoY*. These two isolates are also those that are most distantly related from the rest of the isolates in this population.

**FIG 4:**
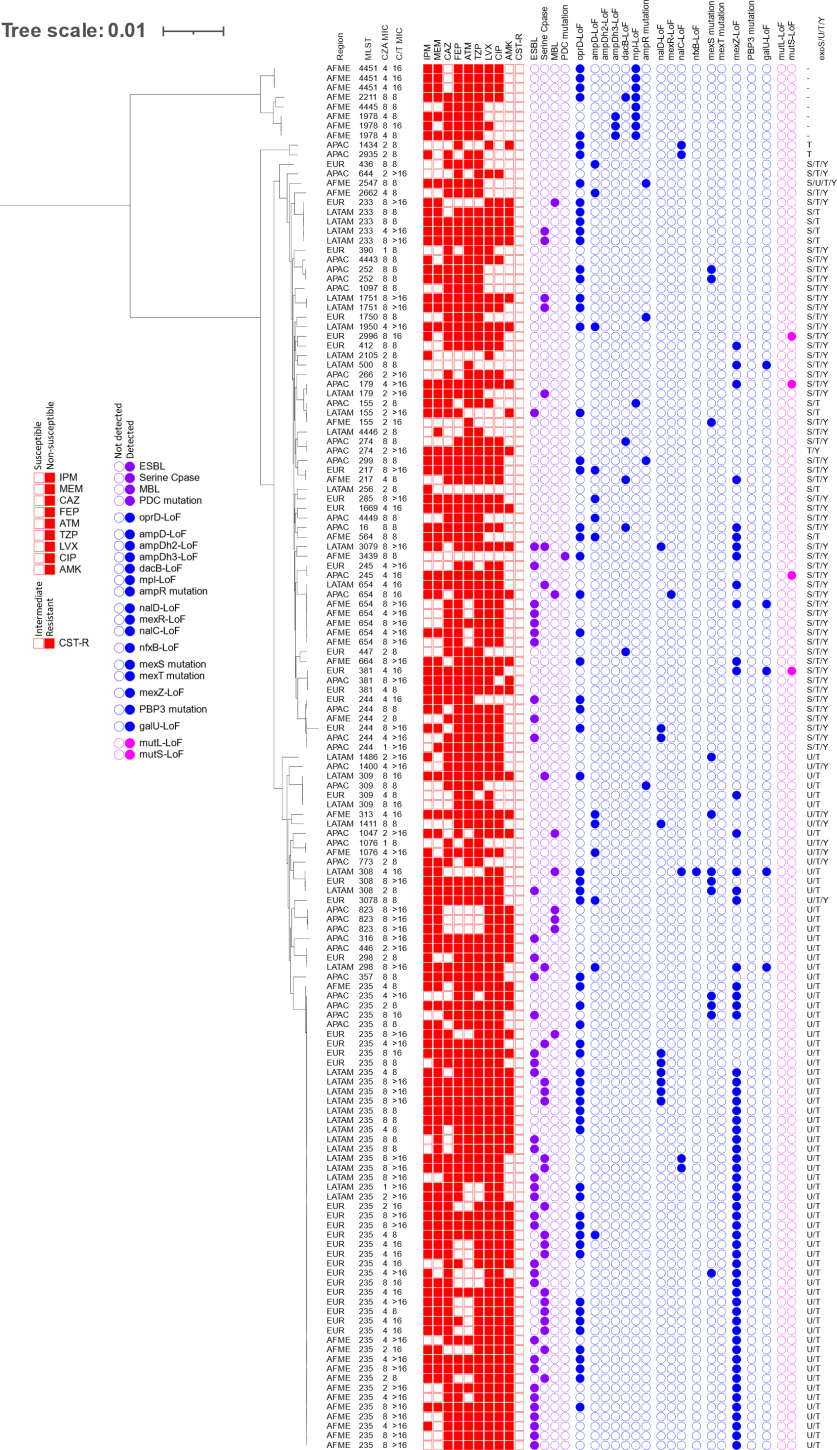
Molecular characteristics and antibiogram of CZA-S and C/T-NS isolates. CZA, ceftazidime-avibactam; C/T, ceftolozane-tazobactam; IPM, imipenem; MEM, meropenem; CAZ, ceftazidime; FEP, cefepime; ATM, aztreonam; TZP, piperacillin-tazobactam; LVX, levofloxacin; CIP, ciprofloxacin; AMK, amikacin; CST-R, colistin-resistant; AFME, Africa/Middle East; APAC, Asia/Pacific; EUR, Europe; LATAM, Latin America; LoF, loss of function. Filled boxes indicate that the isolate tested NS or susceptible, dose dependent (piperacillin-tazobactam only) to the named agent. Filled circles indicate that the named genotype was identified in that isolate. Colors are used to separate antimicrobial susceptibility data (red), enzymatic resistance mechanisms (purple), non-enzymatic resistance mechanisms [blue, (left to right: permeability, ampC upregulation, mexAB-oprM, mexCD-oprJ, mexEF-oprN, mexXY, target mutation, and metabolism)], and genes associated with hypermutation (pink).

A phylogenetic tree of CZA-S and C/T-NS isolates with molecular characteristics and antibiogram is presented in Fig. 4. It is noted that the breakpoints for CZA and C/T differ from one another, and isolates that test with MICs of 8 µg/mL to both agents are categorized as CZA-S and C/T-NS, 30/145 isolates in this population demonstrated this phenotype. Acquired β-lactamases were commonly identified in this population. ESBL genes (not co-carried with MBLs or serine-carbapenemases) were present in 26% of the population, with the majority carrying GES ESBLs. Serine carbapenemase genes were identified in 18% of the isolates and comprised KPC-2, GES-5, and GES-6 carbapenemases. The globally disseminated high-risk clone ST235 was prevalent in this population and most frequently carried ESBLs (24/49 isolates) or serine carbapenemases (17/49 isolates). Approximately, half of the isolates in this population had indicators for upregulation in one or more efflux pumps (*n* = 74) (Fig. S2). One isolate carried both type III secretion effectors *exoS* and *exoU*. A distinct clade was identified containing eight isolates from the region of Africa/Middle East (ST1978, ST2211, ST4445, and ST4451) that do not encode any of *exoS, exoT, exoU,* or *exoY* virulence genes.

**FIG 5:**
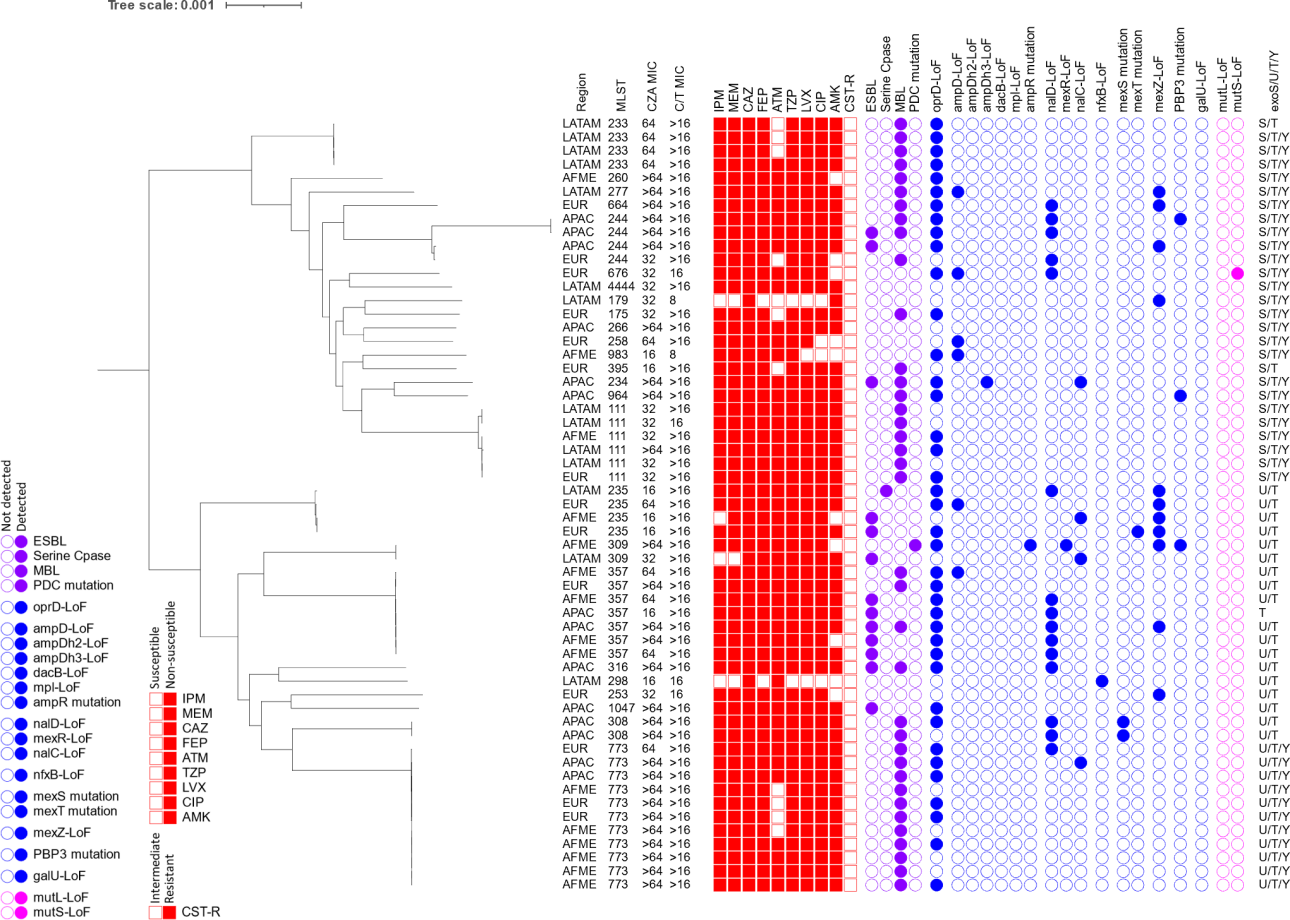
Molecular characteristics and antibiogram of CZA-NS and C/T-NS. CZA, ceftazidime-avibactam; C/T, ceftolozane-tazobactam; IPM, imipenem; MEM, meropenem; CAZ, ceftazidime; FEP, cefepime; ATM, aztreonam; TZP, piperacillin-tazobactam; LVX, levofloxacin; CIP, ciprofloxacin; AMK, amikacin; CST-R, colistin-resistant; AFME, Africa/Middle East; APAC, Asia/Pacific; EUR, Europe; LATAM, Latin America; LoF, loss of function. Filled boxes indicate that the isolate tested NS or susceptible, dose dependent (piperacillin-tazobactam only) to the named agent. Filled circles indicate that the named genotype was identified in that isolate. Colors are used to separate: antimicrobial susceptibility data (red), enzymatic resistance mechanisms (purple), non-enzymatic resistance mechanisms [blue, (left to right: permeability, ampC upregulation, mexAB-oprM, mexCD-oprJ, mexEF-oprN, mexXY, target mutation, and metabolism)], and genes associated with hypermutation (pink).

A phylogenetic tree of CZA-NS and C/T-NS isolates with molecular characteristics and antibiogram is presented in Fig. 5. This population of 57 isolates contained those that were NS to 10/10 agents tested (*n* = 36) and 9/10 agents tested (*n* = 14). MBLs were commonly identified in this subset (*n* = 37, 65%) and included genes encoding VIM, IMP, and NDM. Nearly half of these isolates contained mutations or disruptions in genes that lead to the upregulation of an efflux pump (*n* = 27). Prevalent clones in this group include ST111, ST357, and ST773. Three isolates carried the variants of PBP3 with F533L; two of which also carried an MBL.

## DISCUSSION

In accordance with the *in vitro* activity, CZA and C/T remain as important options for the treatment of infections caused by *P. aeruginosa*. Overall, more isolates were susceptible to CZA than C/T by 1% of those tested. Some resistance mechanisms predominated in isolates susceptible to one agent but not the other. Acquired β-lactamases were identified in a greater proportion of CZA-S and C/T-NS isolates (57.9%) than isolates that were CZA-NS and C/T-S (9.2%), which may be due to a better potentiation of ceftazidime by avibactam relative to the potentiation of ceftolozane by tazobactam. The isolates in this population primarily carried ESBLs (26.2%) or serine-carbapenemases (17.9%), which further supports the role of avibactam, which has the strong inhibitory activity of these β-lactamases. Additionally, more isolates that were CZA-NS and C/T-S carried indicators for MexAB-OprM upregulation and lacked acquired β-lactamases (33%) than isolates that were CZA-S and C/T-NS (2.8%). This is an expected result as ceftazidime is a substrate of MexAB-OprM (39, 40). In addition to MBL carriage, resistance to CZA is found to be mediated by increased efflux via MexAB-OprM upregulation. Regarding other efflux pumps, a greater proportion of isolates that were C/T-NS and CZA-S carried indicators for MexXY upregulation (Fig. S2). Ceftolozane is not known to be a substrate of MexXY, but upregulation is linked to decreased susceptibility to cefepime (41), and 80.6% of isolates in this population were NS to cefepime (Fig. 4). Of the isolates that carried indicators for MexXY upregulation in this population, 82.2% also carried an acquired β-lactamase. As seen in this study, a significant contributor to C/T-resistance is the presence of MBLs, serine carbapenemases, and ESBLs (42). Although not commonly found in the populations in this study, structural variations in PDC are also known to increase hydrolysis of ceftolozane (17–20, 42). It is notable that the (22) breakpoints used in analysis differ for CZA and C/T, and the MICs for both of these agents against this population were similar among a subset of isolates (Table S2). It was also an expected result that MBL-positivity was highest in the population of isolates NS to both agents (64.9%) compared to either other population (≤6%) as neither agent is active against MBL-producing isolates (4, 43).

This study has limitations. Primarily that no population of isolates susceptible to both agents was included. This prevents a comparative analysis on the impact that individual resistance mechanisms have on the agents investigated. For example, there could be isolates that carry ESBLs or hyperproduce MexAB-OprM that are susceptible to both agents, but this study would not identify those. Additionally, this study did not directly measure gene expression, which could be of particular importance with regards to efflux pumps and AmpC. This likely contributed to the lack of any detected resistance mechanism among 30% of isolates that were CZA-NS and C/T-S. As these are surveillance data of non-consecutive isolates, information pertaining to antimicrobial therapy on patients or emergence of resistance over the course of treatment was not available.

This study corroborates that resistance mechanisms for C/T and CZA in *P. aeruginosa* do not completely overlap. It also shows the frequency of resistance mechanisms in globally collected populations of resistant isolates. Noting the differences underlying resistance to these two agents underscores the importance of continuous surveillance for changes in the prevalence of resistance mechanisms such as ESBLs and MBLs at both the global and regional levels to help predict the continued viability of important antipseudomonal agents and to the guide the future development of novel antimicrobial agents.

## Data Availability

All draft genomic assemblies have been deposited at DDBJ/ENA/GenBank under BioProject ID PRJNA1147740.

## SUPPLEMENTAL MATERIAL

Supplemental material aac.00670-24-s0001.docx Tables S1 to S4; Figures S1 and S2.

ASM does not own the copyrights to Supplemental Material that may be linked to, or accessed through, an article. The authors have granted ASM a non-exclusive, world-wide license to publish the Supplemental Material files. Please contact the corresponding author directly for reuse.
