# Supplementary material for: Molecular characterization of clinically isolated Pseudomonas aeruginosa with varying resistance to ceftazidime-avibactam and ceftolozane-tazobactam collected as a part of the ATLAS global surveillance program from 2020 to 2021
Source: Antimicrob Agents Chemother. 2024 Sep 10;68(10):e00670-24. doi: 10.1128/aac.00670-24 (PMC11459925; doi:10.1128/aac.00670-24)
Supplement: Supplemental material — Tables S1 to S4; Figures S1 and S2. [file aac.00670-24-s0001.docx]

**Supplementary Table 1. β-lactamase variants by country, MLST, and phenotype**

| **Region (n characterized)** | **Country** | **MLST** | **Non-intrinsic β-lactamases** | **Phenotype** | **N** |
| --- | --- | --- | --- | --- | --- |
| Africa (30) | Côte d Ivoire | 773 | NDM-1 | C/T-NS, CZA-R | 1 |
|  | Morocco | 235 | GES-1 | C/T-NS, CZA-S | 2 |
|  |  |  | GES-5 | C/T-NS, CZA-S | 1 |
|  |  | 773 | NDM-1 | C/T-NS, CZA-R | 1 |
|  |  |  | NDM-1; OXA-46-New variant; VIM-2 | C/T-NS, CZA-R | 1 |
|  |  | 1978 | - | C/T-S, CZA-R | 1 |
|  |  | Novel | OXA-10 | C/T-NS, CZA-S | 3 |
|  | Nigeria | 235 | GES-1 | C/T-NS, CZA-R | 1 |
|  |  |  | GES-1 | C/T-NS, CZA-S | 8 |
|  |  |  | GES-5 | C/T-NS, CZA-S | 1 |
|  |  | 244 | CTX-M-15; SCO-1; TEM-1 | C/T-NS, CZA-S | 1 |
|  |  | 773 | NDM-1 | C/T-NS, CZA-R | 2 |
|  |  | 1978 | - | C/T-NS, CZA-S | 1 |
|  | South Africa | 111 | VIM-2 | C/T-NS, CZA-R | 1 |
|  |  | 155 | - | C/T-NS, CZA-S | 1 |
|  |  | 235 | OXA-10-New variant | C/T-NS, CZA-S | 1 |
|  |  | 357 | L1-Type; LCR-1 | C/T-NS, CZA-R | 1 |
|  |  |  | VEB-1 | C/T-S, CZA-R | 1 |
|  |  | 773 | NDM-1 | C/T-NS, CZA-R | 1 |
| Asia (67) | Hong Kong | 244 | - | C/T-NS, CZA-S | 1 |
|  |  |  | - | C/T-S, CZA-R | 1 |
|  |  | 252 | - | C/T-NS, CZA-S | 2 |
|  |  | 274 | - | C/T-S, CZA-R | 1 |
|  |  | 313 | - | C/T-S, CZA-R | 1 |
|  |  | 1284 | - | C/T-S, CZA-R | 1 |
|  | India | 9 | - | C/T-S, CZA-R | 2 |
|  |  | 234 | NDM-1; OXA-10; PME-1 | C/T-NS, CZA-R | 1 |
|  |  | 244 | IMP-1; LCR-1; NDM-1; OXA-10; OXA-129; PAU-1; PME-1; TEM-1 | C/T-NS, CZA-R | 1 |
|  |  |  | LCR-1; NDM-1; OXA-10; OXA-129; PAU-1; PME-Trunc | C/T-NS, CZA-R | 1 |
|  |  |  | - | C/T-NS, CZA-S | 1 |
|  |  |  | - | C/T-S, CZA-R | 1 |
|  |  | 266 | - | C/T-NS, CZA-R | 1 |
|  |  |  | - | C/T-NS, CZA-S | 1 |
|  |  | 274 | - | C/T-NS, CZA-S | 1 |
|  |  | 308 | NDM-1; OXA-10 | C/T-NS, CZA-R | 1 |
|  |  | 316 | GES-1 | C/T-NS, CZA-S | 1 |
|  |  |  | OXA-10; VEB-1; VIM-11 | C/T-NS, CZA-R | 1 |
|  |  | 357 | NDM-1; OXA-10; VEB-14 | C/T-NS, CZA-R | 1 |
|  |  |  | OXA-10; VEB-1 | C/T-NS, CZA-R | 1 |
|  |  | 639 | - | C/T-S, CZA-R | 2 |
|  |  | 654 | VIM-2 | C/T-NS, CZA-S | 1 |
|  |  | 773 | NDM-1 | C/T-NS, CZA-R | 1 |
|  |  | 823 | VIM-2 | C/T-NS, CZA-S | 3 |
|  |  | 1047 | NDM-1; OXA-10; VIM-18 | C/T-NS, CZA-S | 1 |
|  |  |  | OXA-10; PME-1 | C/T-NS, CZA-R | 1 |
|  |  | 1076 | - | C/T-NS, CZA-S | 1 |
|  |  | 1434 | - | C/T-NS, CZA-S | 1 |
|  | Japan | 155 | - | C/T-NS, CZA-S | 1 |
|  |  | 299 | - | C/T-NS, CZA-S | 1 |
|  |  | 348 | - | C/T-S, CZA-R | 1 |
|  | Korea, South | 235 | OXA-10 | C/T-NS, CZA-S | 1 |
|  |  | 244 | - | C/T-S, CZA-R | 1 |
|  |  | 245 | - | C/T-S, CZA-R | 1 |
|  |  | 357 | LCR-1 | C/T-S, CZA-R | 1 |
|  |  | 773 | NDM-1; OXA-796; | C/T-NS, CZA-R | 1 |
|  |  | Novel | - | C/T-S, CZA-R | 1 |
|  | Malaysia | 155 | - | C/T-S, CZA-R | 1 |
|  |  | 244 | CARB-2; OXA-10; VEB-3 | C/T-NS, CZA-S | 1 |
|  |  | 261 | - | C/T-S, CZA-R | 1 |
|  |  | 308 | NDM-1 | C/T-NS, CZA-R | 1 |
|  |  | 465 | - | C/T-S, CZA-R | 1 |
|  |  | 2935 | - | C/T-NS, CZA-S | 1 |
|  |  | Novel | - | C/T-NS, CZA-S | 1 |
|  | Taiwan | 274 | - | C/T-NS, CZA-S | 1 |
|  |  | 357 | VEB-1 | C/T-NS, CZA-S | 1 |
|  |  | 381 | - | C/T-S, CZA-R | 1 |
|  |  | 612 | - | C/T-S, CZA-R | 1 |
|  |  | 644 | - | C/T-NS, CZA-S | 1 |
|  |  | 1400 | - | C/T-NS, CZA-S | 1 |
|  |  | Novel | - | C/T-NS, CZA-S | 1 |
|  | Thailand | 179 | - | C/T-NS, CZA-S | 1 |
|  |  |  | - | C/T-S, CZA-R | 1 |
|  |  | 235 | OXA-10 | C/T-NS, CZA-S | 1 |
|  |  | 244 | OXA-10; VEB-New variant | C/T-NS, CZA-R | 1 |
|  |  | 381 | - | C/T-NS, CZA-S | 1 |
|  |  | 964 | IMP-6; NDM-1 | C/T-NS, CZA-R | 1 |
|  |  | 1076 | - | C/T-S, CZA-R | 1 |
|  |  | 1097 | - | C/T-NS, CZA-S | 1 |
|  |  | 2012 | - | C/T-S, CZA-R | 1 |
|  |  | 3277 | - | C/T-S, CZA-R | 1 |
|  |  | 3426 | - | C/T-S, CZA-R | 1 |
| Europe (76) | Belgium | 235 | GES-1 | C/T-NS, CZA-S | 3 |
|  |  | 446 | - | C/T-S, CZA-R | 1 |
|  |  | 560 | - | C/T-S, CZA-R | 1 |
|  |  | Novel | - | C/T-S, CZA-R | 1 |
|  | Czech Republic | 235 | GES-5 | C/T-NS, CZA-S | 4 |
|  |  | 244 | - | C/T-S, CZA-R | 1 |
|  |  | 357 | IMP-7; OXA-2 | C/T-NS, CZA-R | 1 |
|  | France | 175 | - | C/T-S, CZA-R | 1 |
|  |  | 258 | - | C/T-NS, CZA-R | 1 |
|  |  | 308 | - | C/T-NS, CZA-S | 1 |
|  |  | 390 | - | C/T-NS, CZA-S | 1 |
|  |  | 2410 | - | C/T-S, CZA-R | 1 |
|  |  | 2996 | - | C/T-NS, CZA-S | 1 |
|  | Germany | 309 | - | C/T-NS, CZA-S | 1 |
|  |  | 446 | - | C/T-S, CZA-R | 1 |
|  |  | 646 | - | C/T-S, CZA-R | 1 |
|  | Greece | 235 | VIM-2 | C/T-NS, CZA-S | 1 |
|  |  | 395 | OXA-10; VIM-2 | C/T-NS, CZA-R | 1 |
|  |  | 640 | - | C/T-S, CZA-R | 1 |
|  |  | 676 | - | C/T-NS, CZA-R | 1 |
|  | Hungary | 217 | - | C/T-NS, CZA-S | 1 |
|  |  | 348 | - | C/T-S, CZA-R | 1 |
|  |  | 395 | - | C/T-S, CZA-R | 1 |
|  |  | 1750 | - | C/T-NS, CZA-S | 1 |
|  | Ireland | 235 | - | C/T-S, CZA-R | 1 |
|  | Italy | 179 | - | C/T-S, CZA-R | 1 |
|  |  | 235 | GES-1 | C/T-NS, CZA-R | 1 |
|  |  |  | - | C/T-NS, CZA-R | 1 |
|  |  | 381 | - | C/T-NS, CZA-S | 1 |
|  |  | 447 | - | C/T-NS, CZA-S | 1 |
|  |  | 876 | - | C/T-S, CZA-R | 1 |
|  |  | 1710 | - | C/T-S, CZA-R | 1 |
|  | Latvia | 235 | SHV-12 | C/T-NS, CZA-S | 1 |
|  |  | 705 | - | C/T-S, CZA-R | 1 |
|  | Lithuania | 245 | GES-7 | C/T-NS, CZA-S | 1 |
|  |  | 298 | OXA-17 | C/T-NS, CZA-S | 1 |
|  |  |  | - | C/T-S, CZA-R | 1 |
|  | Poland | 253 | - | C/T-NS, CZA-R | 1 |
|  |  | 313 | - | C/T-S, CZA-R | 1 |
|  |  | 436 | - | C/T-NS, CZA-S | 1 |
|  | Portugal | 235 | GES-6 | C/T-NS, CZA-S | 1 |
|  |  | 244 | - | C/T-S, CZA-R | 1 |
|  |  |  | - | C/T-NS, CZA-S | 1 |
|  |  |  | - | C/T-S, CZA-R | 1 |
|  | Romania | 233 | VIM-2 | C/T-NS, CZA-S | 1 |
|  |  | 235 | GES-5 | C/T-NS, CZA-S | 1 |
|  | Slovenia | 111 | VIM-2 | C/T-NS, CZA-R | 1 |
|  | Spain | 175 | OXA-2; VIM-20 | C/T-NS, CZA-R | 1 |
|  |  | 235 | GES-5 | C/T-NS, CZA-S | 1 |
|  |  | 244 | OXA-2; PER-1 | C/T-NS, CZA-S | 1 |
|  |  |  | VIM-2 | C/T-NS, CZA-R | 1 |
|  |  |  | - | C/T-S, CZA-R | 1 |
|  |  | 285 | - | C/T-NS, CZA-S | 1 |
|  |  | 296 | - | C/T-S, CZA-R | 1 |
|  |  | 381 | - | C/T-NS, CZA-S | 1 |
|  |  | 412 | - | C/T-NS, CZA-S | 1 |
|  |  | Novel | - | C/T-S, CZA-R | 1 |
|  | Switzerland | 170 | - | C/T-S, CZA-R | 1 |
|  | Turkey | 235 | GES-5 | C/T-NS, CZA-S | 3 |
|  |  |  | OXA-17 | C/T-NS, CZA-S | 1 |
|  |  | 773 | OXA-10; VIM-2 | C/T-NS, CZA-R | 1 |
|  |  | 3078 | - | C/T-NS, CZA-S | 1 |
|  | Ukraine | 235 | GES-1 | C/T-NS, CZA-S | 2 |
|  |  | 386 | - | C/T-S, CZA-R | 1 |
|  |  | 664 | VIM-1 | C/T-NS, CZA-R | 1 |
|  |  | 773 | NDM-1 | C/T-NS, CZA-R | 2 |
|  |  | 1669 | - | C/T-NS, CZA-S | 1 |
| Latin America (75) | Argentina | 233 | OXA-4; VIM-2 | C/T-NS, CZA-R | 2 |
|  |  | 235 | OXA-1; OXA-129 | C/T-NS, CZA-S | 3 |
|  |  |  | OXA-1; OXA-129 | C/T-S, CZA-R | 4 |
|  |  |  | OXA-1; OXA-129; OXA-17 | C/T-S, CZA-R | 1 |
|  |  |  | OXA-129; OXA-17 | C/T-NS, CZA-S | 2 |
|  |  | 1411 | - | C/T-NS, CZA-S | 1 |
|  |  | 1639 | - | C/T-S, CZA-R | 1 |
|  | Brazil | 233 | OXA-4; VIM-2 | C/T-NS, CZA-R | 1 |
|  |  | 235 | CTX-M-2; OXA-129 | C/T-NS, CZA-S | 1 |
|  |  |  | CTX-M-229 | C/T-NS, CZA-S | 3 |
|  |  |  | KPC-2; OXA-1 | C/T-NS, CZA-S | 1 |
|  |  |  | KPC-2; OXA-1; OXA-129 | C/T-NS, CZA-R | 1 |
|  |  |  | KPC-2; OXA-1; OXA-129 | C/T-NS, CZA-S | 2 |
|  |  |  | - | C/T-S, CZA-R | 1 |
|  |  | 245 | - | C/T-S, CZA-R | 1 |
|  |  | 256 | - | C/T-NS, CZA-S | 1 |
|  |  | 277 | OXA-396; OXA-56; SPM-1 | C/T-NS, CZA-R | 1 |
|  |  | 298 | - | C/T-NS, CZA-R | 1 |
|  |  | 1560 | - | C/T-S, CZA-R | 3 |
|  |  | 2060 | - | C/T-S, CZA-R | 1 |
|  |  | 3079 | GES-1; KPC-2 | C/T-NS, CZA-S | 1 |
|  |  | 3701 | - | C/T-S, CZA-R | 1 |
|  | Chile | 111 | VIM-2 | C/T-NS, CZA-R | 2 |
|  |  | 235 | - | C/T-S, CZA-R | 1 |
|  |  | 309 | - | C/T-NS, CZA-S | 1 |
|  |  | 395 | CARB-2 | C/T-S, CZA-R | 1 |
|  |  | 654 | KPC-2 | C/T-NS, CZA-S | 1 |
|  |  | 1486 | - | C/T-NS, CZA-S | 1 |
|  |  | 1950 | - | C/T-NS, CZA-S | 1 |
|  | Colombia | 111 | - | C/T-S, CZA-R | 1 |
|  |  | 179 | KPC-2 | C/T-NS, CZA-S | 1 |
|  |  | 298 | CARB-2; KPC-2; OXA-2 | C/T-NS, CZA-S | 1 |
|  |  | 1751 | KPC-2 | C/T-NS, CZA-S | 2 |
|  |  | 3044 | - | C/T-S, CZA-R | 1 |
|  | Guatemala | 233 | KPC-2; OXA-10; OXA-4; TEM-Trunc | C/T-NS, CZA-S | 1 |
|  |  |  | KPC-2; OXA-4; TEM-Trunc | C/T-NS, CZA-S | 1 |
|  |  |  | OXA-10 | C/T-NS, CZA-S | 2 |
|  |  | 235 | KPC-2; TEM-Trunc | C/T-NS, CZA-S | 2 |
|  |  |  | OXA-2 | C/T-S, CZA-R | 1 |
|  |  | Novel | - | C/T-NS, CZA-S | 1 |
|  |  |  | - | C/T-S, CZA-R | 1 |
|  | Mexico | 155 | GES-1; OXA-2 | C/T-NS, CZA-S | 1 |
|  |  | 233 | OXA-4; VIM-2 | C/T-NS, CZA-R | 1 |
|  |  | 235 | - | C/T-S, CZA-R | 1 |
|  |  | 244 | - | C/T-S, CZA-R | 1 |
|  |  | 275 | - | C/T-S, CZA-R | 2 |
|  |  | 308 | GES-1 | C/T-NS, CZA-S | 1 |
|  |  | 309 | GES-5; OXA-2 | C/T-NS, CZA-S | 1 |
|  |  |  | GES-19; GES-26 | C/T-NS, CZA-R | 1 |
|  |  | 2105 | - | C/T-NS, CZA-S | 1 |
|  |  | Novel | - | C/T-NS, CZA-R | 1 |
|  | Panama | 111 | IMP-18; OXA-2 | C/T-NS, CZA-R | 1 |
|  |  | 168 | - | C/T-S, CZA-R | 1 |
|  |  | 179 | OXA-2-New variant | C/T-NS, CZA-R | 1 |
|  | Venezuela | 111 | VIM-2 | C/T-NS, CZA-R | 1 |
|  |  | 308 | VIM-2 | C/T-NS, CZA-S | 1 |
|  |  | 500 | - | C/T-NS, CZA-S | 1 |
|  |  | 1930 | - | C/T-S, CZA-R | 1 |
| Middle East (44) | Israel | 155 | - | C/T-S, CZA-R | 1 |
|  |  | 217 | - | C/T-NS, CZA-S | 1 |
|  |  | 260 | - | C/T-S, CZA-R | 1 |
|  |  | 313 | - | C/T-NS, CZA-S | 1 |
|  |  | 571 | - | C/T-S, CZA-R | 1 |
|  |  | 644 | - | C/T-S, CZA-R | 1 |
|  |  | 654 | GES-1 | C/T-NS, CZA-S | 5 |
|  |  | 1076 | - | C/T-NS, CZA-S | 1 |
|  |  | 1097 | - | C/T-S, CZA-R | 1 |
|  |  | 1753 | - | C/T-S, CZA-R | 1 |
|  |  | 2105 | - | C/T-S, CZA-R | 1 |
|  |  | 2211 | - | C/T-NS, CZA-S | 1 |
|  | Jordan | 381 | - | C/T-S, CZA-R | 1 |
|  |  | 664 | OXA-256 | C/T-NS, CZA-S | 1 |
|  | Kuwait | 12 | - | C/T-S, CZA-R | 1 |
|  |  | 270 | - | C/T-S, CZA-R | 1 |
|  |  | 274 | - | C/T-S, CZA-R | 1 |
|  |  | 357 | OXA-10; VEB-New variant | C/T-NS, CZA-R | 1 |
|  |  |  | VEB-1 | C/T-NS, CZA-R | 1 |
|  |  | 381 | - | C/T-S, CZA-R | 1 |
|  |  | 557 | - | C/T-S, CZA-R | 1 |
|  |  | 676 | - | C/T-S, CZA-R | 1 |
|  |  | 2547 | - | C/T-NS, CZA-S | 1 |
|  |  | 3439 | - | C/T-NS, CZA-S | 1 |
|  |  | Novel | - | C/T-S, CZA-R | 2 |
|  | Qatar | 162 | - | C/T-S, CZA-R | 1 |
|  |  | 309 | - | C/T-NS, CZA-R | 1 |
|  |  |  | - | C/T-S, CZA-R | 1 |
|  |  | 357 | VEB-1 | C/T-NS, CZA-R | 1 |
|  |  | 564 | - | C/T-NS, CZA-S | 1 |
|  |  | 639 | - | C/T-S, CZA-R | 1 |
|  |  | 983 | - | C/T-NS, CZA-R | 1 |
|  |  | 1978 | - | C/T-NS, CZA-S | 1 |
|  |  | 2662 | - | C/T-NS, CZA-S | 1 |
|  |  | Novel | - | C/T-S, CZA-R | 1 |
|  | Saudi Arabia | 170 | - | C/T-S, CZA-R | 1 |
|  |  | 260 | IMP-1; OXA-10 | C/T-NS, CZA-R | 1 |
|  |  | 1978 | - | C/T-NS, CZA-S | 1 |
|  |  | Novel | - | C/T-NS, CZA-S | 1 |
| South Pacific (8) | Australia | 16 | - | C/T-NS, CZA-S | 1 |
|  |  | 155 | - | C/T-S, CZA-R | 1 |
|  |  | 245 | - | C/T-NS, CZA-S | 1 |
|  |  | 309 | - | C/T-NS, CZA-S | 1 |
|  |  | 446 | - | C/T-NS, CZA-S | 1 |
|  | Philippines | 235 | CTX-M-15; OXA-1 | C/T-NS, CZA-S | 1 |
|  |  |  | - | C/T-NS, CZA-S | 1 |
|  |  | 773 | - | C/T-NS, CZA-S | 1 |

Supplementary Table 2. Frequency distribution of ceftazidime-avibactam and ceftolozane-tazobactam MIC values against characterized isolates of *P. aeruginosa*, by phenotypic category.

| **Phenotype** | **N** | **Agent** | **MIC (µg/mL)** | | | | | | | | |
| --- | --- | --- | --- | --- | --- | --- | --- | --- | --- | --- | --- |
|  |  |  | **0.5** | **1** | **2** | **4** | **8** | **16** | **32** | **64** | **>endpoint** |
| C/T-NS, CZA-NS | 57 | C/T |  |  |  |  | 2 | 4 |  |  | **51** |
|  |  | CZA |  |  |  |  |  | 7 | 12 | 10 | **28** |
| C/T-NS, CZA-S | 145 | C/T |  |  |  |  | 56 | 27 |  |  | **62** |
|  |  | CZA |  | 4 | 26 | 44 | **71** |  |  |  |  |
| C/T-S, CZA-NS | 98 | C/T | 1 | 9 | 26 | **62** |  |  |  |  |  |
|  |  | CZA |  |  |  |  |  | **80** | 14 | 4 |  |

Modal values are in bold

Abbreviations: CZA, ceftazidime-avibactam; C/T, ceftolozane-tazobactam; S, susceptible; NS, nonsusceptible; >endpoint, the MIC exceeded the highest concentrations tested for ceftazidime-avibactam (64 µg/mL) or ceftolozane-tazobactam (16 µg/mL).

Supplementary Figure 1. Proportion of carbapenemases detected, by gene, and proportion of ESBLs detected in carbapenemase-negative isolates.

AfME, Africa/Middle East; APAC, Asia Pacific; EUR, Europe; LATAM, Latin America. *GES variants: GES-1, GES-5, GES-6, GES-7, GES-19, GES-26. IMP variants: IMP-1, IMP-6, IMP-7, IMP-18. VIM variants: VIM-1, VIM-2, VIM-11, VIM-18, VIM-20. CTX-M variants: CTX-M-2. CTX-M-15, CTX-M-229. VEB variants: VEB-1, VEB-3, VEB-14, VEB-new variant.

Supplementary Figure 2. Proportion of efflux upregulation, by indicators, detected in each phenotypic category.

Abbreviations: CZA, ceftazidime-avibactam; C/T, ceftolozane-tazobactam; S, susceptible; NS, nonsusceptible

Supplementary Table 3. Mutations analyzed in genes of interest

| **Gene name** | **Mutation(s) of interest** | **Interpretation** | **Reference** |
| --- | --- | --- | --- |
| *oprD* | Loss/truncation | ΔoprD | (26) |
| *blaPDC* | T96I, F147L, Q157R, G183D, G242R, E247K/G, N373I, Del G229-E247, or Del T316-A319 | PDC(e)-[mutation] | (17, 18, 19, 20, 42) |
| *ampD* | P41S, H77Y, R82C, G84D, F89S, C92Y, A96T, G121R, T139A, P162L, or R164S, Loss/truncation | PDC↑ | (5, 12) |
| *ampDh2* | Loss/truncation | PDC↑ | (5) |
| *ampDh3* | Loss/truncation | PDC↑ | (5) |
| *dacB (pbp4)* | Loss/truncation | PDC↑ | (5) |
| *mpl* | Loss/truncation | PDC↑ | (6) |
| *ampR* | D135N or G154R | PDC↑ | (14, 15, 16) |
| *nalD* | Loss/truncation | MexAB-OprM↑ | (7) |
| *mexR* | Loss/truncation | MexAB-OprM↑ | (7) |
| *nalC* | Loss/truncation | MexAB-OprM↑ | (7) |
| *mexZ* | Loss/truncation | MexXY↑ | (8) |
| *nfxB* | Loss/truncation | MexCD-oprJ↑ | (9) |
| *mexS* | D44E, S60P/F, A166P, F185L, L263Q, V73A+L270Q, Loss/truncation | MexEF-oprN↑ | (11) |
| *mexT* | G257S/A | MexEF-oprN↑ | (10) |
| *pbp3 (ftsI)* | Y503C/H F533L | PBP3-[mutation] | (27, 28) |
| *galU* | Loss/truncation | Aminoglycoside, cephalosporin resistance | (29, 30, 31) |
| *mutL* | P159S and H288Y, K310M, DEL341 "Q", R631C, Loss/truncation | Hypermutator phenotype | (34, 35, 36) |
| *mutS* | C224R, T287P, Loss/truncation | Hypermutator phenotype | (34, 35, 36) |

Supplementary Table 4. Efflux upregulation indicators, *ampC* upregulation indicators, and known PDC mutations among isolates that carried only intrinsic β-lactamases by phenotypic category.

| **Phenotype (N)** | **Efflux upregulation indicators; pump(s) impacted** | **ampC upregulation indicators** | **Known PDC mutations** | **N** |
| --- | --- | --- | --- | --- |
| CZA-NS, C/T-NS (9) | None | None |  | 2 |
|  |  | ampD |  | 2 |
|  | mexR, mexZ; mexAB-oprM, mexXY | ampR | N373I | 1 |
|  | mexZ; mexXY | None |  | 1 |
|  |  | ampD |  | 1 |
|  | nalD; mexAB-oprM | ampD |  | 1 |
|  | nfxB; mexCD-oprJ | None |  | 1 |
| CZA-S, C/T-NS (61) | None | None |  | 22 |
|  |  | ampD |  | 8 |
|  |  | ampDh3, mpl |  | 3 |
|  |  | ampR |  | 4 |
|  |  | dacB |  | 2 |
|  |  | dacB, mpl |  | 1 |
|  |  | mpl |  | 2 |
|  | mexS; mexEF-oprN | None |  | 4 |
|  | mexZ, mexS; mexXY, mexEF-oprN | None |  | 1 |
|  | mexZ; mexXY | None | DEL229-247 "GYGKDDRPLRVGPGPLDAE" | 1 |
|  |  | None |  | 5 |
|  |  | ampD |  | 2 |
|  |  | dacB |  | 2 |
|  | nalC; mexAB-oprM | None |  | 2 |
|  | nalD; mexAB-oprM | None |  | 1 |
|  |  | ampD |  | 1 |
| CZA-NS, C/T-S (89) | None | None |  | 29 |
|  |  | ampD |  | 14 |
|  |  | ampDh3, mpl |  | 1 |
|  |  | ampR |  | 1 |
|  |  | dacB |  | 2 |
|  | mexR, mexZ; mexAB-oprM, mexXY | None |  | 1 |
|  | mexR; mexAB-oprM | None | F147L;E247K | 1 |
|  |  | None |  | 12 |
|  |  | ampD |  | 2 |
|  | mexZ; mexXY | None |  | 7 |
|  |  | ampD |  | 3 |
|  | nalC; mexAB-oprM | None |  | 3 |
|  | nalD, mexZ; mexAB-oprM, mexXY | None |  | 1 |
|  | nalD, nalC, mexZ; mexAB-oprM, mexXY | ampD |  | 1 |
|  | nalD; mexAB-oprM | None |  | 6 |
|  |  | ampD |  | 4 |
|  |  | dacB |  | 1 |
